# Prenatal detection of novel compound heterozygous variants of the *PLD1* gene in a fetus with congenital heart disease

**DOI:** 10.3389/fgene.2024.1498485

**Published:** 2024-11-01

**Authors:** Linyan Zhu, Mei Chen, Yubo Shi, Xiaxi Huang, Huiqing Ding

**Affiliations:** ^1^ Department of Obstetrics and Gynaecology, The First Affiliated Hospital of Ningbo University, Ningbo, Zhejiang, China; ^2^ Women’s Hospital, School of Medicine, Zhejiang University, Hangzhou, Zhejiang, China

**Keywords:** *PLD1*, congenital heart disease, prenatal diagnosis, whole-exome sequencing, rare dieseases

## Abstract

**Background:**

Congenital heart disease (CHD) is the most common birth defect and heart valve defects are the most common cardiac defect, accounting for over 25% of all congenital heart diseases. To date, more than 400 genes have been linked to CHD, the genetic analysis of CHD cases is crucial for both clinical management and etiological determination. Patients with autosomal-recessive variants of *PLD1* are predisposed to Cardiac Valvular Dysplasia-1 (CVDP1), which predominantly affects the right-sided heart valves, including the pulmonic, tricuspid, and mitral valves.

**Methods:**

Databases were utilized to predict the impact of the c.1062-59A>G variant on splicing. Whole-exome sequencing (WES), reverse transcription polymerase chain reaction (RT-PCR), Sanger sequencing, and TA clone sequencing were conducted on both the parents and the fetus.

**Results:**

A compound heterozygous variation in the *PLD1*(NM_002662.5):c.1937G>C (p. G646A) from the father and *PLD1*(NM_002662.5):c.1062-59A>G from the mother, was identified and confirmed in the fetus. The c.1937G>C (p. G646A) and the c.1062-59A>G variants were all classified as variant of uncertain significance (VUS) per ACMG guidelines. RT-PCR and TA clone sequencing revealed a 76-bp intronic insertion and exon 11 skipping in the proband and her mother’s transcripts, causing a frameshift and premature stop codon in *PLD1*. Consequently, after being informed about the risks of their variant of unknown significance (VUS), the couple chose pre-implantation genetic testing for monogenic disorders (PGT-M) and had a healthy child.

**Conclusion:**

Our study identified novel variants to expand the mutation spectrum of CHD and provided reliable evidence for the recurrent risk and reproductive care options.

## 1 Introduction

Congenital heart disease (CHD) encompasses a broad spectrum of structural and functional abnormalities that originate during cardiac embryogenesis (Triedman and Newburger, 2016; Xiao et al., 2024). Globally, CHD affects approximately 7 per 1,000 live births, representing one-third of all congenital anomalies (Dolk et al., 2011; McMahon et al., 2023). The etiology of most congenital heart diseases is attributed to genetic mutations in the embryo, which may be either inherited or *de novo* (Jay et al., 2016; Nees and Chung, 2020). The analysis of *de novo* mutations has highlighted the extensive genetic heterogeneity underlying CHD pathogenesis. To date, it is estimated that over 400 genes contribute to CHD, although only a fraction of these have been fully characterized (Homsy et al., 2015; Narayan et al., 2024; Williams et al., 2019; Zaidi and Brueckner, 2017).

In recent years, the advent and widespread adoption of next-generation sequencing technologies have led to the detection of an unprecedented number of genetic variants. However, our capacity to interpret the functional consequences of these variants, particularly those located outside protein-coding regions, has not kept pace with their discovery (Lord et al., 2024). As a result, around half of patients with rare disorders still do not get a diagnosis (Turro et al., 2020; Stranneheim et al., 2021). Consequently, approximately 50% of patients with rare disorders remain undiagnosed. It has been reported that between 15% and 60% of disease-causing variants impact splicing (Lopez-Bigas et al., 2005). In diagnostic and research variant prioritization pipelines, variants located within the canonical 2 base pair splice acceptor or donor sites are generally classified as splice-affecting, whereas those outside these regions are frequently not. Variants situated in intronic regions outside the canonical splice sites are commonly filtered out, posing challenges for the diagnosis of rare disorders. Therefore, accurately distinguishing between splice-affecting and non-splice-affecting variants is of critical importance.

Phospholipase D1, encoded by the *PLD1* gene, catalyzes the hydrolysis of membrane phosphatidylcholine, resulting in the production of phosphatidic acid. This enzyme activity has been implicated in severe congenital heart valve defects, as documented in five studies (Nelson and Frohman, 2015; Cai et al., 2023; Lahrouchi et al., 2021; Masuda et al., 2023; Ta-Shma et al., 2017). Patients with autosomal-recessive variants of *PLD1* are predisposed to Cardiac Valvular Dysplasia-1 (CVDP1,OMIM 212093), which predominantly affects the right-sided heart valves, including the pulmonic, tricuspid, and mitral valves. Ta-Shma et al. first found that mutations in the *PLD1* gene, either homozygous or compound heterozygous, were linked to cardiac valvular dysplasia (CVDP1) in two unrelated consanguineous families. They observed that structural atrioventricular valve defects were the main issues in patients with *PLD1* loss-of-function. During early embryonic heart development, some endocardial cells on the valvular heart cushions undergo EndoMT, transforming into mesenchymal cells to remodel the extracellular matrix and form valves. Additionally, these individuals exhibit structural cardiac anomalies such as atrial and ventricular septal defects, a single left ventricle, and a hypoplastic right ventricle (Ta-Shma et al., 2017).

In the current investigation, we report a case of fetus with pulmonary atresia, tricuspid valve dysplasia and significant tricuspid regurgitation of a non-consanguineous couple. Prenatal whole-exome sequencing followed by Sanger sequencing identified two novel mutations in the *PLD1* gene.

## 2 Methods

### 2.1 Subject

**FIGURE 1:**
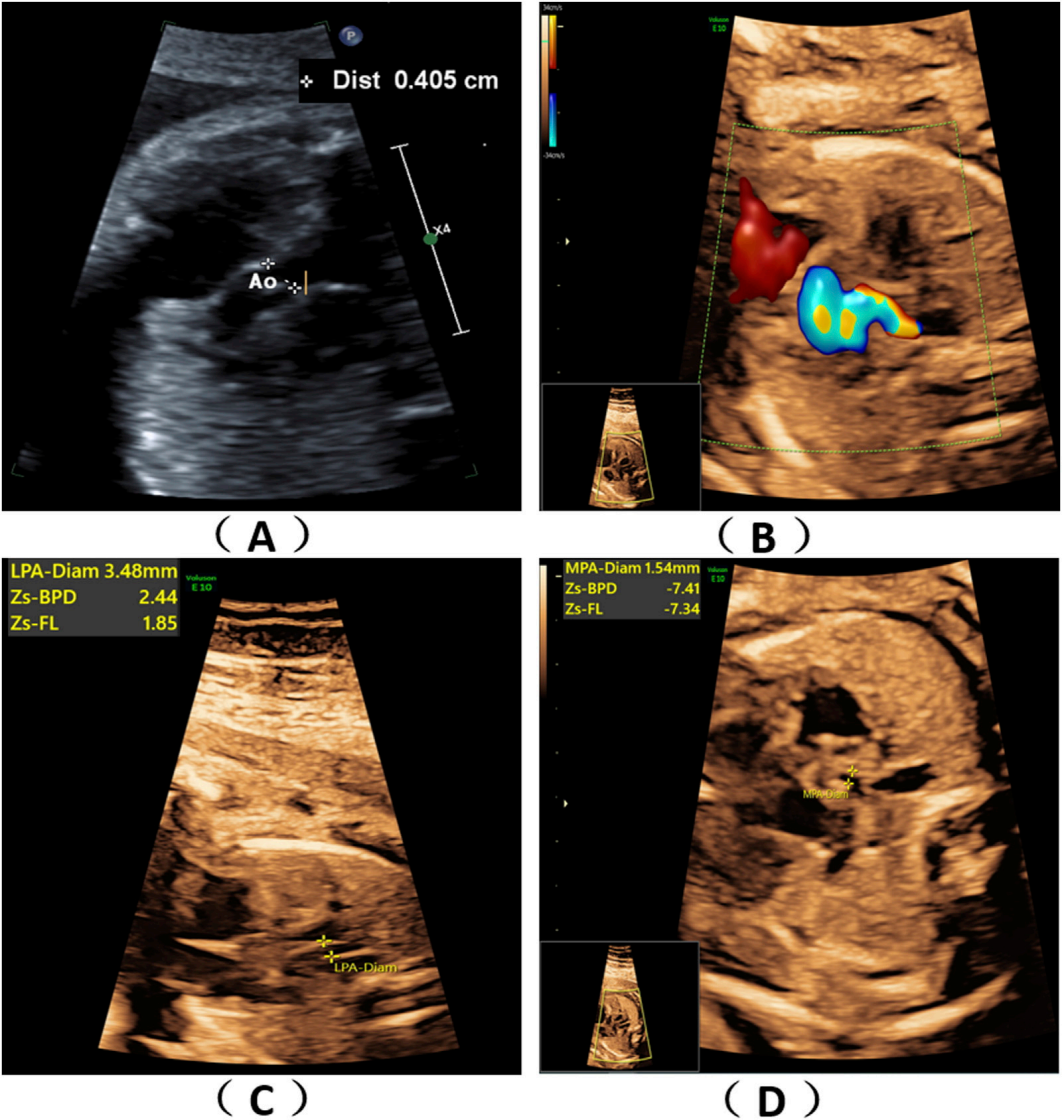
Fetal ultrasound scan findings. Ultrasound scans of the fetus showed fetal congenital heart disease including pulmonary atresia, regurgitation and tricuspid valve dysplasia at the 24th week of gestation **(A–D)**.

A 36-year-old healthy woman was referred to our clinic. Two years ago, her first pregnancy was terminated at 24th week with a right absent radius and ventricular septal defect and genetic tests (karyotype and chromosomal microarray analysis) were normal, but WES was not performed. The woman was already on her second pregnancy at 24 weeks of gestation when she came to our hospital. Ultrasound scans of the fetus showed fetal congenital heart disease including pulmonary atresia, regurgitation and tricuspid valve dysplasia at the 24th week of gestation (Figures 1A–D). To explore the genetic cause, amniocentesis was performed at 24^+1^ weeks of gestational age. The pregnancy was terminated at 27^+5^th week after the fetal genetic diagnosis and thorough genetic counseling. This study was approved by the Ethics Committee of The First Affiliated Hospital of Ningbo University and conformed to the Declaration of Helsinki. All participants provided their written informed consents.

### 2.2 Biological sampling and DNA extraction

Amniocentesis was conducted at the 24th week of gestation. A total of 20 mL of amniotic fluid from the fetus and 5 mL of peripheral blood from the parentswere collected for DNA extraction utilizing the QIAamp DNA Blood Mini Kit (Qiagen, Germany). The extracted DNA was subsequently quantified using the NanoDrop 2000 spectrophotometer (Thermo Fisher Scientific). After induced labor of the fetus, we obtained 5 mL of umbilical cord blood to extract the total RNA.

### 2.3 Whole‐exome sequencing (WES)

Whole-exome sequencing (WES) was conducted on an Illumina HiSeq2000 platform (Illumina) following the manufacturer’s protocol. Clean reads, each with a minimum length of 90 base pairs, derived from targeted sequencing and subsequent filtering, were aligned to the human genome reference (hg19) utilizing the Burrows-Wheeler Aligner (BWA) Multi-Vision software package (Li and Durbin, 2009). Analysis of sequencing coverage and depth within the target region, as well as the identification of single-nucleotide variants (SNVs) and insertions/deletions (indels), was performed using the Genome Analysis Toolkit (GATK) (McKenna et al., 2010). Variants were filtered by population databases, including the Genome Aggregation Database. Next, the output files were used to perform sequencing coverage and depth analysis of the target region, single-nucleotide variants (SNVs), and indel calling, the GATK software was used to detect SNVs and indels Variants were filtered by population databases.

To predict the pathogenicity of the mutations, SIFT (http://sift.jcvi.org) and Mutation Taster (http://www.MutationTaster.org) were mainly used. The variants were interpreted under the guideline of the American College of Medical Genetics and Genomics (ACMG) (Richards et al., 2015) and the Association for Molecular Pathology (AMP), with only those variants pertinent to the proband’s clinical manifestations being documented. The patient’s genomic sequence was aligned with the GRCh37 (hg19) human genome reference sequence, and all potential pathogenic variants were subsequently validated through Sanger sequencing methods.

### 2.4 Sanger sequencing

Sanger sequencing was carried out to validate the variants. The primers for c.1937G>C were 5′- AGG​ACT​GTA​AAA​CCT​TGT​TAG​CCC​A -3′and 5′- CAA​AAA​TCA​ACG​GAA​GCA​AAA​CAA -3′. The primers for c.1062-59A>G were 5′- CAG​GAT​AGG​AAA​ATT​AAT​CTG​GCT​C -3′and 5′- CAC​CAG​TCT​GTG​ATA​AAA​ATC​TCT​T -3′, with the PCR protocol template: 95°C, 10min; then 35 cycles of 94°C,30s,60°C,30s and 72°C 30s; then 72°C,10min. Products were sequenced with the ABI 3730 DNA analyzer (Applied Biosystems).

### 2.5 Total RNA extraction, reverse transcription polymerase chain reaction (RT-PCR), TA cloning and sequencing analysis

Total RNA was extracted from the fetal umbilical cord blood and peripheral blood of the parents with TaKaRaMiniBEST Universal RNA Extraction Kit (TaKaRa, Japan) according to the manufacturer’s instructions. Complementary DNA (cDNA) was synthesized using the PrimeScript 1st strand cDNA Synthesis Kit (TaKaRa, Japan). The PCR primers were 5′- GGG​AAG​AGC​CTG​CTA​CAG​AT -3′and 5′- ATT​GAT​GCC​AAG​AGC​GAG​TT -3′. The PCR products were separated by electrophoresis on a 1.5% agarose gel. Minimal differences were observed between the two mRNA splicing products, complicating subsequent sequencing efforts. To address this issue, TA cloning was employed to purify the PCR products using the HieffClone™ Zero TOPO-TA Cloning Kit (Yeasen, China). Following purification, the selected PCR product was subjected to sequencingon an ABI 3500xL Dx Genetic Analyzer (Applied Biosystems, United States).

## 3 Results

### 3.1 Compound heterozygous variants of *PLD1* gene

**FIGURE 2:**
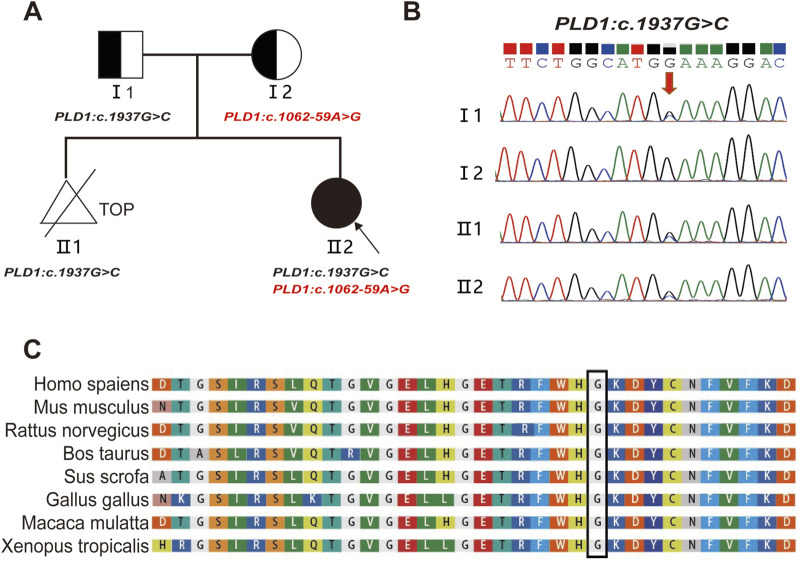
Pedigree and the analysis of the variant c.1937G>C in the *PLD1* gene. **(A)** Pedigree of the family and the compound heterozygous mutations in the *PLD1* gene. TOP: Termination Of Pregnancy **(B)** Sanger sequencing analysis. The variant c.1937G>C was validated by Sanger sequencing (Red arrows indicated the mutation). **(C)** Conservation status among orthologs of the c.1937G.

The compound heterozygous variants of *PLD1*: c.1937G>C p. (G646A) and c.1062-59A>G were identified in the fetus (Ⅱ2) by whole-exome sequencing. Sanger sequencing confirmed that the two variants were inherited from the father c.1937G>C (p. G646A) (Figures 2A, B) and the mother c.1062-59A>G (Figure 4A), respectively. The evolutionary conservation of the p.(G646A) amino acid was highly conserved across 8 species (Figure 2C).The gnomAD database records the allele frequency of c.1937G>C p. (G646A) variant in the East Asian population as 0.0004010 (PM2); it is not listed in the HGMD database, and the ClinVar database rates it as of uncertain significance; MutationTaster, PolyPhen-2, and M-CAP all predict it as pathogenic/likely pathogenic (PP3); According to the ACMG guidelines (2015), the c.1937G>C p. (G646A) variant is classified as variant of uncertain significance (VUS).

### 3.2 Pathogenicity of *PLD1*: c.1062-59A>G

**FIGURE 3:**
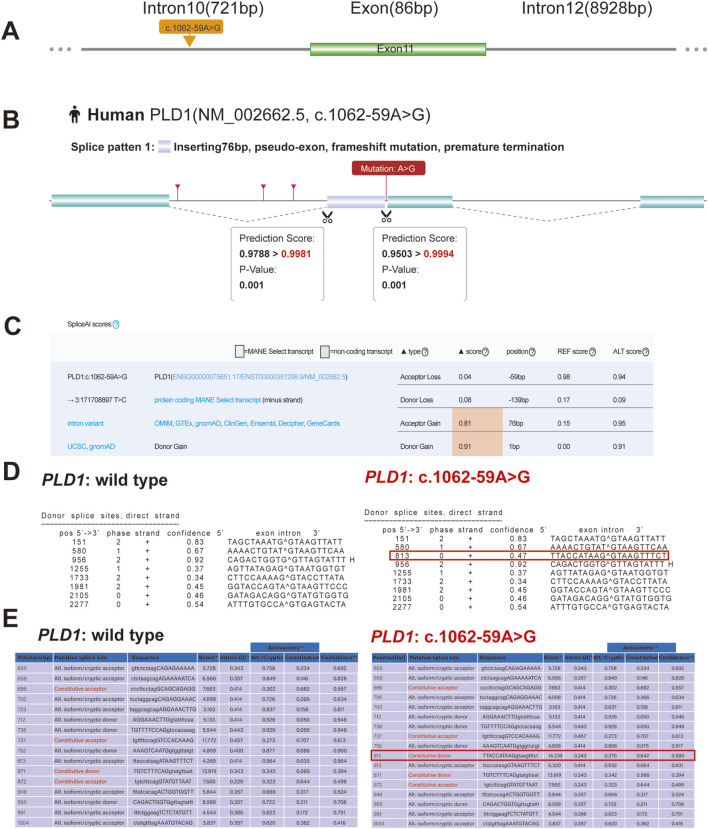
Predictive results of the (C)9212-6T > G variant site in splicing. **(A)** Yellow arrow indicates location of the c.9212-6T > G variant. **(B)** RDDC^SC^ Predicts that c.1062-59A>G variant of *PLD1* gene affects splicing by inserting 76bp intron. **(C)** The effect of the c.1062-59A>G variant creates a new acceptor splice site and a new donor splice site, which is predicted with SpliceAI. **(D)** The Predictive result of wild type and c.1062-59A>G variant of *PLD1* by using NetGene2 Server shows that the c.1062-59A>G variant creates a new donor splice site. **(E)** The Predictive result shows that c.1062-59A>G variant of *PLD1* also creates a new donor splice site by using ASSP tool.

**FIGURE 4:**
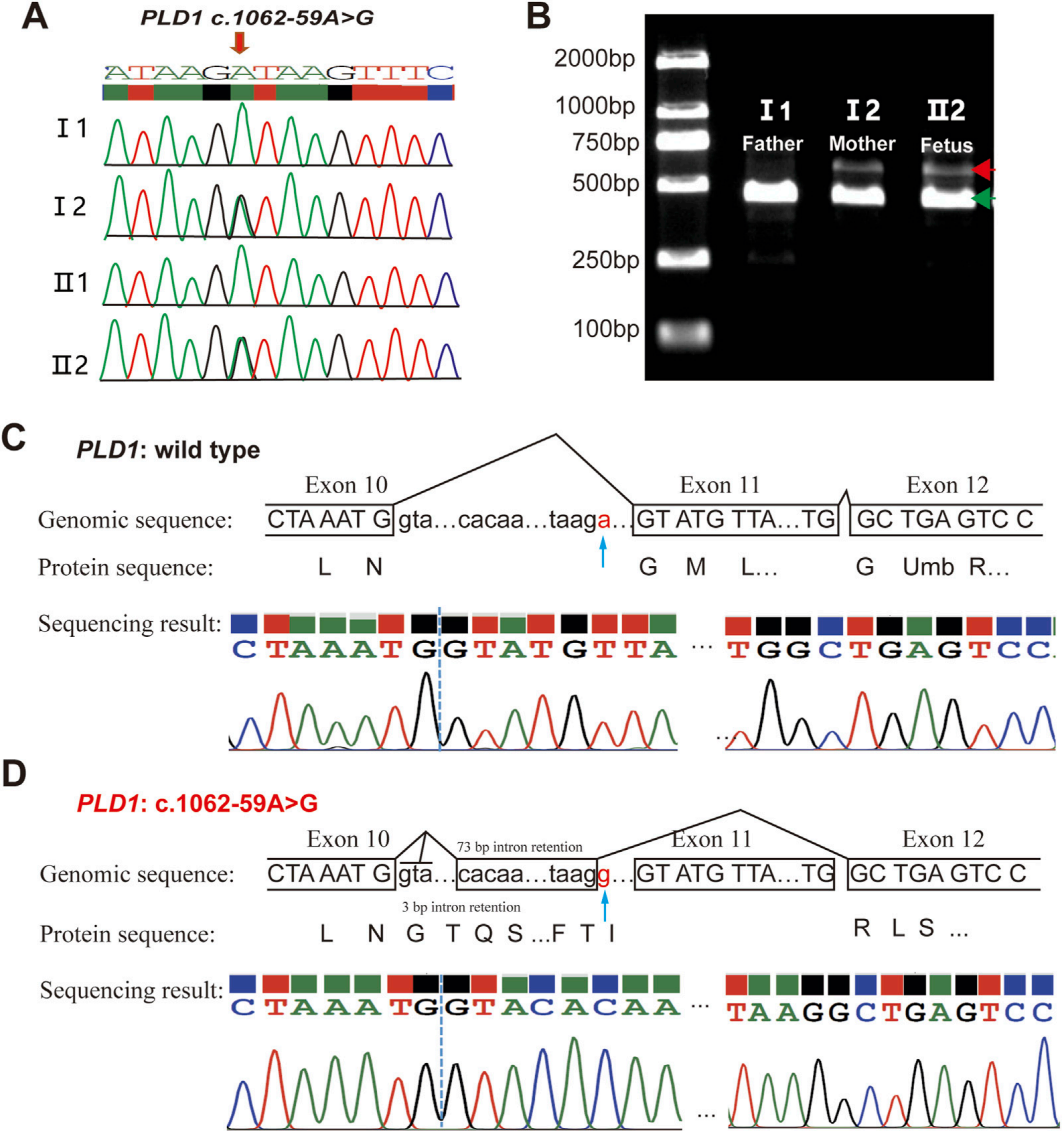
Analysis of the c.1062-59A>G variant in the *PLD1* gene. **(A)** Sanger sequencing analysis. The variant c.1062-59A>G was validated by Sanger sequencing. **(B)** RT-PCR analysis of *PLD1* cDNA from the family members. Agarose gel (1.5%) electrophoresis of RT-PCR products generated from I1(father), I2(mother)and II2(the proband). Aberrantly spliced mRNA and normal spliced mRNA are marked by red and green arrowheads, respectively. **(C, D)** TA clone sequencing and analysis of the RT-PCR products from the proband. The blue arrows reveal the position of the c.1062-59A>G variant. *PLD1*: c.1062-59A>G is truncated compared with wild type *PLD1*.

The variant c.1062-59A>G is located in intron10 (Figure 3A). RDDC^SC^ (https://rddc.tsinghua-gd.org/zh/tool/rna-splicer) (Figure 3B), SpliceAI (https://spliceailookup.broadinstitute.org/) (Figure 3C), NetGene2 Server (http://www.cbs.dtu.dk/services/NetGene2/) (Figure 3D) and Alternative Splice Site Predictor (ASSP) (http://wangcomputing.com/assp/index.html) (Figure 3E) were used to predict the effect of the variant c.1062-59A>G on splicing. Results showed that the variant affected splicing. For the validation, RNAs were extracted from the fetus and the couple. cDNAs were transcribed to amplify exons 9–13 of *PLD1* with the primers. 1.5% agarose gel electrophoresis demonstrated that the fetus and the mother had aberrantly spliced mRNA comparing with the normal mRNA (Figure 4B). TA clone sequencing and sequencing showed that the c.1062-59A>G variant caused 76-bp intron retention and the skipping of exon 11 (Figures 4C, D), leading to the loss function of *PLD1* gene. Considering the accordance between phenotype and genotype and the rarity of the splicing variant, the mutation *PLD1*: c.1062-59A>G was also classified as variant of uncertain significance (VUS) according to ACMG guidelines.

## 4 Discussion

In the current investigation, ultrasound scans of the first fetus (Ⅱ1) in the family showed the absence of the right forearm radius bone and ventricular septal defect, chromosomal karyotype and CMA (chromosomal microarray analysis) did not show any apparent abnormalities. The pregnancy was terminated at 24th week. Two years later, the woman got second pregnancy, ultrasound scans of the fetus showed that pulmonary atresia, regurgitation and tricuspid valve dysplasia at the 24th week of gestation. WES testing identified that the fetus (Ⅱ2) had compound heterozygous variants of *PLD1*: c.1937G>C p. (G646A) and c.1062-59A>G inheriting from the parents. Based on ACMG criteria, both c.1937G>C p. (G646A) and c.1062-59A>G were classified as variant of uncertain significance (VUS).

With the highly genotype-phenotype correlation and predicted results of RDDC^SC^, SpliceAI, NetGene2 Server and ASSP, RT-PCR and TA clone sequencing were performed to investigate the pathology of the splicing mutation. The c.1062-59A>G variant led to 76-bp intron insertion and the skipping of Exon11. The evolutionary conservation of the p.(G646A) amino acid was highly conserved across 8 species. Even based on these data, the pathogenicity of the two VUS sites still cannot be upgraded to likely pathogenic. Given the consistent genotype and phenotype, we suspect that the compound heterozygous variation in *PLD1* might cause CHD in the fetus. Further functional experiments are needed to confirm its impact on *PLD1* protein expression and enzyme activity.

Sanger sequencing showed that the first fetus had the missense mutation c.1937G>C, but no c.1062-59A>G variant. Unfortunately, due to insufficient DNA quantity, WES testing was not able to perform on the first fetus. So, it is unclear whether the phenotype of the first fetus is caused by mutations in genes other than the *PLD1* gene. It brings confusion to the couple’s genetic counseling for the future pregnancy.

Due to the complexity and heterogeneity of clinical presentations in CHD, newborns are often susceptible to under diagnosis at birth, which can even lead to fatality (Martin et al., 2022; Thomford et al., 2018; Wren et al., 2008). In developed countries, more than 75% of children with coronary heart disease can survive into adulthood, including those with complex abnormalities (Welch et al., 2023; Lopez et al., 2020). Furthermore, coronary heart disease may be associated with identifiable genetic syndromes (Fahed et al., 2013; Shabana et al., 2020; Armendariz et al., 2023) with the continuous progress in genomic technology and animal models, we are gradually unraveling the etiology of these diseases. Specifically, mutations in the *PLD1* gene are responsible for congenital valvular diseases, a severe cardiac condition known as Cardiac Valvular Dysplasia-1 (CVDP1). Building on previous research, considering *PLD1* as a disease gene is expected to facilitate reproductive counseling and pre-implantation genetic screening for affected families.

*PLD1* gene is located on the chromosome 3q26.31, consisting of 27 exons. In 2017, it was first reported that the variants of *PLD1* gene were associated with the cardiac valvular defect (Ta-Shma et al., 2017). Lahrouchi et al. (2021) identified 30 patients with *PLD1* variants who presented predominantly with congenital cardiac valve defects. Cai et al. (2023) identified novel variants of *PLD1* gene in a Chinese family with recurrent fetal congenital heart defects. Theses published work related to *PLD1* gene strongly supports the pathogenic role of these variants in CHD.

This 36-year-old couple has experienced two fetal malformations and missed the ideal childbearing age. They are determined to conceive again and request PGT-M. If the disease-causing gene is identified, PGT-M can help prevent recurrence in their children. However, unclear gene pathogenicity and variants of unknown significance (VUS) can lead to misdiagnosis and complicate genetic counseling. At present, the lack of guidelines in the United States has given rise to disparities among PGT - M laboratories. Genetic counselors in laboratories advocate the application of PGT - M for conditions with VUS, on the condition that informed consent is obtained (Porto et al., 2022). We conducted a follow-up with this couple, who, after providing informed consent regarding potential risks, underwent third-generation *in vitro* fertilization at a different medical facility. They have since successfully had a healthy child.

In summary, we described a fetus diagnosed as cardiac valvular dysplasia and identified two novel variants of *PLD1* gene via WES and Sanger sequencing. Our findings expand the mutation spectrum and provided information for the genetic counseling of cardiac valvular dysplasia.

## Data Availability

The datasets for this article are not publicly available due to concerns regarding participant/patient anonymity. Requests to access the datasets should be directed to the corresponding author.

## References

[B1] Armendariz D. A. Sundarrajan A. Hon G. C. (2023). Breaking enhancers to gain insights into developmental defects. Elife 12, 12. 10.7554/elife.88187 PMC10374278 37497775

[B2] Cai R. Tan Y. Wang M. Yu H. Wang J. Ren Z. (2023). Detection of novel pathogenic variants in two families with recurrent fetal congenital heart defects. Pharmgenomics Pers. Med. 16, 173–181. 10.2147/PGPM.S394120 36923242 PMC10008912

[B3] Dolk H. Loane M. Garne E. European Surveillance of Congenital Anomalies EUROCAT Working Group (2011). Congenital heart defects in Europe: prevalence and perinatal mortality, 2000 to 2005. Circulation 123 (8), 841–849. 10.1161/CIRCULATIONAHA.110.958405 21321151

[B4] Fahed A. C. Gelb B. D. Seidman J. G. Seidman C. E. (2013). Genetics of congenital heart disease: the glass half empty. Circ. Res. 112 (4), 707–720. 10.1161/CIRCRESAHA.112.300853 23410880 PMC3827691

[B5] Homsy J. Zaidi S. Shen Y. Ware J. S. Samocha K. E. Karczewski K. J. (2015). *De novo* mutations in congenital heart disease with neurodevelopmental and other congenital anomalies. Science 350 (6265), 1262–1266. 10.1126/science.aac9396 26785492 PMC4890146

[B6] Jay P. Y. Akhirome E. Magnan R. A. Zhang M. R. Kang L. Qin Y. (2016). Transgenerational cardiology: one way to a baby's heart is through the mother. Mol. Cell Endocrinol. 435, 94–102. 10.1016/j.mce.2016.08.029 27555292 PMC5014674

[B7] Lahrouchi N. Postma A. V. Salazar C. M. De Laughter D. M. Tjong F. Piherová L. (2021). Biallelic loss-of-function variants in *PLD1* cause congenital right-sided cardiac valve defects and neonatal cardiomyopathy. J. Clin. investigation 131 (5), e142148. 10.1172/JCI142148 PMC7919725 33645542

[B8] Li H. Durbin R. (2009). Fast and accurate short read alignment with Burrows-Wheeler transform. Bioinformatics 25 (14), 1754–1760. 10.1093/bioinformatics/btp324 19451168 PMC2705234

[B9] Lopez K. N. Morris S. A. Sexson Tejtel S. K. Espaillat A. Salemi J. L. (2020). US mortality attributable to congenital heart disease across the lifespan from 1999 through 2017 exposes persistent racial/ethnic disparities. Circulation 142 (12), 1132–1147. 10.1161/CIRCULATIONAHA.120.046822 32795094 PMC7508797

[B10] Lopez-Bigas N. Audit B. Ouzounis C. Parra G. Guigo R. (2005). Are splicing mutations the most frequent cause of hereditary disease? FEBS Lett. 579 (9), 1900–1903. 10.1016/j.febslet.2005.02.047 15792793

[B11] Lord J. Oquendo C. J. Wai H. A. Douglas A. G. L. Bunyan D. J. Wang Y. (2024). Predicting the impact of rare variants on RNA splicing in CAGI6. Hum. Genet. 10.1007/s00439-023-02624-3 PMC11976748 38170232

[B12] Martin G. R. Schwartz B. N. Hom L. A. Donofrio M. T. (2022). Lessons learned from infants with late detection of critical congenital heart disease. Pediatr. Cardiol. 43 (3), 580–585. 10.1007/s00246-021-02760-5 34709442

[B13] Masuda Y. Nagayasu Y. Murakami H. Nishie R. Morita N. Hashida S. (2023). Triple repeated fetal congenital heart disease linked to *PLD1* mutation: a case report. J. Med. Case Rep. 17 (1), 411. 10.1186/s13256-023-04149-9 37770978 PMC10540367

[B14] McKenna A. Hanna M. Banks E. Sivachenko A. Cibulskis K. Kernytsky A. (2010). The Genome Analysis Toolkit: a MapReduce framework for analyzing next-generation DNA sequencing data. Genome Res. 20 (9), 1297–1303. 10.1101/gr.107524.110 20644199 PMC2928508

[B15] McMahon C. J. Voges I. Jenkins P. Brida M. van der Bosch A. E. Dellborg M. (2023). Adult congenital heart disease training in Europe: current status, disparities and potential solutions. Open Heart 10 (2), e002558. 10.1136/openhrt-2023-002558 38097363 PMC10729203

[B16] Narayan P. Richter F. Morton S. (2024). Genetics and etiology of congenital heart disease. Curr. Top. Dev. Biol. 156, 297–331. 10.1016/bs.ctdb.2024.01.009 38556426

[B17] Nees S. N. Chung W. K. (2020). Genetic basis of human congenital heart disease. Cold Spring Harb. Perspect. Biol. 12 (9), a036749. 10.1101/cshperspect.a036749 31818857 PMC7280080

[B18] Nelson R. K. Frohman M. A. (2015). Physiological and pathophysiological roles for phospholipase D. J. Lipid Res. 56 (12), 2229–2237. 10.1194/jlr.R059220 25926691 PMC4655994

[B19] Porto A. Gaber Caffrey R. Crowley-Matoka M. Spencer S. Li M. Propst L. (2022). Offering preimplantation genetic testing for monogenic disorders (PGT-M) for conditions with reduced penetrance or variants of uncertain significance: ethical insight from U.S. laboratory genetic counselors. J. Genet. Couns. 31 (1), 261–268. 10.1002/jgc4.1482 34347921

[B20] Richards S. Aziz N. Bale S. Bick D. Das S. Gastier-Foster J. (2015). Standards and guidelines for the interpretation of sequence variants: a joint consensus recommendation of the American College of medical genetics and genomics and the association for molecular pathology. official J. Am. Coll. Med. Genet. 17 (5), 405–424. 10.1038/gim.2015.30 PMC4544753 25741868

[B21] Shabana N. A. Shahid S. U. Irfan U. (2020). Genetic contribution to congenital heart disease (CHD). Pediatr. Cardiol. 41 (1), 12–23. 10.1007/s00246-019-02271-4 31872283

[B22] Stranneheim H. Lagerstedt-Robinson K. Magnusson M. Kvarnung M. Nilsson D. Lesko N. (2021). Integration of whole genome sequencing into a healthcare setting: high diagnostic rates across multiple clinical entities in 3219 rare disease patients. Genome Med. 13 (1), 40. 10.1186/s13073-021-00855-5 33726816 PMC7968334

[B23] Ta-Shma A. Zhang K. Salimova E. Zernecke A. Sieiro-Mosti D. Stegner D. (2017). Congenital valvular defects associated with deleterious mutations in the *PLD1* gene. J. Med. Genet. 54 (4), 278–286. 10.1136/jmedgenet-2016-104259 27799408

[B24] Thomford N. E. Dzobo K. Yao N. A. Chimusa E. Evans J. Okai E. (2018). Genomics and epigenomics of congenital heart defects: expert review and lessons learned in Africa. OMICS 22 (5), 301–321. 10.1089/omi.2018.0033 29762087 PMC6016577

[B25] Triedman J. K. Newburger J. W. (2016). Trends in congenital heart disease: the next decade. Circulation 133 (25), 2716–2733. 10.1161/CIRCULATIONAHA.116.023544 27324366

[B26] Turro E. Astle W. J. Megy K. Gräf S. Greene D. Shamardina O. (2020). Whole-genome sequencing of patients with rare diseases in a national health system. Nature 583 (7814), 96–102. 10.1038/s41586-020-2434-2 32581362 PMC7610553

[B27] Welch C. L. Aldred M. A. Balachandar S. Dooijes D. Eichstaedt C. A. Gräf S. (2023). Defining the clinical validity of genes reported to cause pulmonary arterial hypertension. Genet. Med. official J. Am. Coll. Med. Genet. 25 (11), 100925. 10.1016/j.gim.2023.100925 PMC10766870 37422716

[B28] Williams K. Carson J. Lo C. (2019). Genetics of congenital heart disease. Biomolecules 9 (12), 879. 10.3390/biom9120879 31888141 PMC6995556

[B29] Wren C. Reinhardt Z. Khawaja K. (2008). Twenty-year trends in diagnosis of life-threatening neonatal cardiovascular malformations. Arch. Dis. Child. Fetal Neonatal Ed. 93 (1), F33–F35. 10.1136/adc.2007.119032 17556383

[B30] Xiao F. Zhang X. Morton S. U. Kim S. W. Fan Y. Gorham J. M. (2024). Functional dissection of human cardiac enhancers and noncoding *de novo* variants in congenital heart disease. Nat. Genet. 56 (3), 420–430. 10.1038/s41588-024-01669-y 38378865 PMC11218660

[B31] Zaidi S. Brueckner M. (2017). Genetics and genomics of congenital heart disease. Circ. Res. 120 (6), 923–940. 10.1161/CIRCRESAHA.116.309140 28302740 PMC5557504

